# Ethylene Synthesis and Regulated Expression of Recombinant Protein in *Synechocystis* sp. PCC 6803

**DOI:** 10.1371/journal.pone.0050470

**Published:** 2012-11-21

**Authors:** Fernando Guerrero, Verónica Carbonell, Matteo Cossu, Danilo Correddu, Patrik R. Jones

**Affiliations:** University of Turku, Department of Biochemistry and Food Chemistry, Turku, Finland; University of New South Wales, Australia

## Abstract

The ethylene-forming enzyme (EFE) from *Pseudomonas syringae* catalyzes the synthesis of ethylene which can be easily detected in the headspace of closed cultures. A synthetic codon-optimized gene encoding N-terminal His-tagged EFE (EFEh) was expressed in *Synechocystis* sp. PCC 6803 (*Synechocystis*) and *Escherichia coli* (*E. coli*) under the control of diverse promoters in a self-replicating broad host-range plasmid. Ethylene synthesis was stably maintained in both organisms in contrast to earlier work in *Synechococcus elongatus* PCC 7942. The rate of ethylene accumulation was used as a reporter for protein expression in order to assess promoter strength and inducibility with the different expression systems. Several metal-inducible cyanobacterial promoters did not function in *E. coli* but were well-regulated in cyanobacteria, albeit at a low level of expression. The *E. coli* promoter P_trc_ resulted in constitutive expression in cyanobacteria regardless of whether IPTG was added or not. In contrast, a Lac promoter variant, P_A1lacO-1_, induced EFE-expression in *Synechocystis* at a level of expression as high as the Trc promoter and allowed a fine level of IPTG-dependent regulation of protein-expression. The regulation was tight at low cell density and became more relaxed in more dense cultures. A synthetic quorum-sensing promoter system was also constructed and shown to function well in *E. coli*, however, only a very low level of EFE-activity was observed in *Synechocystis*, independent of cell density.

## Introduction

Genetically tractable cyanobacteria are gaining attention as a host for the direct photosynthetic conversion of sunlight and CO_2_ into chemical energy. Recently, different strains of cyanobacteria have been engineered for the production of hydrogen [1], ethylene [2]–[4], ethanol [5], butanol [6], isoprene [7] and fatty acids [8]. The complete genome of >50 cyanobacteria species is available and comprehensive stoichiometric reconstructions have been developed [9]. However, until now there are few reports of comprehensive metabolic engineering of multi-step pathways [8], [10]. In order to enable economically sustainable biological conversion of solar energy, H_2_O and CO_2_ into fuel it is necessary to engineer the catalytic hosts specifically for the intended biotechnological purpose. This requires a molecular toolbox for metabolic engineering including promoters for user-regulated protein expression.

Promoters are the DNA regions that control gene expression and contribute to determine the rate of transcription and therefore the quantity of protein that is synthesized. In bacteria the transcription is initiated when the promoter sequence is recognized by a sigma (σ) factor which allows the formation of the RNA polymerase (RNAP) holoenzyme [11]. Currently there is a vast quantity of standard biological parts and devices described [12] that has primarily been characterized in *E. coli*. The performance of diverse promoters in cyanobacteria was recently reviewed by Heidorn and coworkers [13].

For biotechnological purposes it may be important to regulate the expression of the gene(s) of interest, particularly if the gene products, their catalyzed metabolism or potentially toxic metabolites has a negative effect on growth [14]. Well-repressed promoters may also be important in fundamental studies where the impact from the expression of a particular protein is studied with specific timing. Typically used promoters in cyanobacteria include the strong light inducible psbAI promoter (e.g. [3]), and the RuBisCO subunit rbcLS promoter (e.g. [5]) which are both constitutive under standard growth conditions. Several native metal-inducible promoters from cyanobacteria have been demonstrated to allow fine-tuned control of protein expression, including the copper controlled promoters upstream of the petE (plastocyanin gene) and petJ (cytochrome c553) genes [15], [16], although the relative strength of expression has not been compared with other promoters. Inducible and well-regulated protein expression has been reported with the *E. coli* Trc promoter in *Synechococcus elongatus* PCC 7942 [17], although expression with the Lac promoter was leaky [1]. Recently, Huang and coworkers [18] examined a range of *E. coli* derived systems for protein expression in *Synechocystis* and reached the conclusion that there was no regulated system that was capable of strong expression in this model cyanobacterium.

**Table 1:** Functional blocks used in vector construction and the restriction sites used for their cloning, in the order of 5′ to 3′ ends of each functional block.

Functional block	Restriction sites
Self-replicating region	AvrII – EagI
Selection marker	EagI – BsrGI
Promoter/Regulator	BsrGI – (SpeI) – KpnI
Gene/operon of interest	KpnI – PstI
Transcription terminators	PstI – AvrII

We considered ethylene biosynthesis as metabolic target to (1) establish a model system for photobiological synthesis of volatile hydrocarbon fuel and (2) allow non-invasive monitoring of the performance of engineered expression-systems. There are three known biological pathways for ethylene synthesis [19], [20]. In most organisms the native precursor for ethylene synthesis is methionine. In higher plants ethylene is synthesized from methionine via the intermediate 1-Aminocyclopropane-1-carboxylic acid (ACC) by the enzymes ACC synthase and ACC oxidase [21]. In most prokaryotes, ethylene is formed from methionine via 2-keto-4-methyl-thiobutyric acid (KMBA) catalyzed by an NADH:Fe(III) EDTA oxidoreductase [22]. In a few plant pathogens ethylene is synthesized by an Ethylene Forming Enzyme (EFE) in a complex multi-step reaction utilizing 2-oxoglutarate (2-OG), arginine and dioxygen as substrates [23]. This 2-OG dependent pathway was first reported in *Penicillium digitatum* and has been extensively studied in *Pseudomonas syringae* pv. *phaseolicola* PK2 by Fukuda and coworkers [24], [25]. Heterologous expression of EFE in *E. coli* [24], [26], *Trichoderma viride* [27], *Trichoderma reesei* [28], *Saccharomyces cerevisiae* [29] and the cyanobacteria *Synechococcus elongatus* PCC 7942 [2] and recently in *Synechocystis* sp. PCC 6803 [4] have all resulted in the accumulation of ethylene in the headspace of closed vessels. Curiously, the first cyanobacterial system was highly unstable resulting in rapid development of mutants that lost the capability to synthesize ethylene [3]. Such instability was not reported in any of the other organisms further prompting continued analysis of ethylene-synthesis in cyanobacteria.

**Table 2:** Genetic elements used in this work.

Name	Description	Source
Self-replicating region	RSF1010 derived self-replicating region	pVZ321
Sp/Str	Spectinomycin and streptomycin resistance gene (*aadA*)	pZS43-MCS
LacI^q^	Lac repressor	pTrc99A
P_A1lacO-1_	P_A1lacO-1_ IPTG inducible promoter	pZE13-MCS (Expressys)
P_trc_	IPTG inducible Trc promoter	pTrc99A
P_petE_	P_petE_ Cu^2+^ inducible promoter from plactocyanin	*Synechocystis* sp. PCC 6803
P_coa_	Co^2+^ inducible promoter from coaT gene	*Synechocystis* sp. PCC 6803
P_smt_	Heavy metals inducible promoter from metalothionein	*Synechococcus* sp. PCC 7002
EFEh	Ethylene forming enzyme from *Pseudomonas syringae*, His-tagged	Synthetic, codon optimized for *Synechocystis*
ACS-ACO	ACC synthase and ACC oxidase from *Arabidopsis*	Synthetic, codon optimized for *Synechocystis*
LuxRI	LuxR and LuxI from *Vibrio fischeri*, including the “lux-box”	Synthetic, codon optimized for *Synechocystis*
RhlRI	RhlR and RhlI from *Pseudomonas aeruginosa,* including the intergenic region	Synthetic, codon optimized for *Synechocystis*

**Table 3:** Primers used in this work. Sequences recognized by restriction enzymes are designated in uppercase font. Bases that are not complementary are underlined and overhangs are shown in italic.

Primer	Sequence 5′ − 3′	Additional information
pVZS-F1	Cgcacagctccataggccg	Forward primer near 3′ of self-replicating region
pVZS-R1	gcgcttatggcagagcaggg	Reverse primer near 5′ of self-replicating region
pVZ_Nhe_F	gctgcccggatGCTAGCtgaaagcgacc	Forward primer to amplify self-replicating region
pVZ_Eag_R	gtgacaccacgCGGCCGgcaggagcaga	Reverse primer to amplify self-replicating region
pZS_Eag_F	ggcttaccCGGCCGactgtccctagtgc	Forward primer to amplify Sp/Str resistance cassette
pTrc_Avr_Rev	cagggttattgtCCTAGGagcggatac	Reverse primer to amplify repressor and promoter region in pDF-trc-EFEh
pTrc_Bsr_Fw	ctgacgggctTGTACActcccggcatc	Forward primer to amplify repressor and promoter region in pDF-trc-EFEh
pSp1_Rev	catcaaacatcgacccacggcg	Forward primer to control insertions
pZE13_Spe_F	cagggttattgACTAGTgagcggatac	Forward primer to clone Lac promoter from pZE13-MCS vector to prepare pDF-lac-EFEh
pZE13_Kpn_R	gggggcccGGTACCtttctcctct	Reverse primer to clone Lac promoter from pZE13-MCS vector
petE_Bsr_Fw	*cagtTGTACA*agcggttgcccaatctaac	Forward primer to clone petE promoter from 6803 genome
petE_Eco_Rev	*catgGAATTC*atacttcttggcgattg	Reverse primer to clone petE promoter from 6803 genome
coaR_Bsr_Fw	*cagtTGTACA*gcacactaaagacaagtgag	Forward primer to clone coaR repressor and promoter from 6803 genome
coaR_Eco_R	*tctcGAATTC*gctttttaacttggatttttacc	Reverse primer to clone coaR repressor and promoter from 6803 genome
smtA_Bsr_Fw	*atatTGTACA*ttagtgggtgtgtccatcctc	Forward primer to clone smtA repressor and promoter from 7002 genome
smtA_Eco_R	*gtgtGAATTC*ctattggtgacaagtacagccc	Reverse primer to clone smtA repressor and promoter from 7002 genome
pDF_Spe_Fw	gcaacgcaattaatACTAGTtagcgcgaattgatc	Generate SpeI site by site directed mutagenesis
pDF_Spe_Rev	gatcaattcgcgctaACTAGTattaattgcgttgc	Generate SpeI site by site directed mutagenesis

## Materials and Methods

### 1.1 Organisms and growth conditions

*Escherichia coli* strain DH5α was grown routinely in LB medium at 37°C. Liquid cultures were incubated in a rotary shaker at 150–200 rpm. LB was supplemented with 1.5% (w/v) bactoagar for solid cultures and FeCl_3_ (where indicated). The growth medium was supplemented with the appropriate antibiotics at the following concentrations: 50 µg/ml spectinomycin (Sp), 20 µg/ml streptomycin (Str), 100 µg/ml ampicillin (Amp). *Synechocystis* sp. PCC 6803 (glucose-tolerant strain, hereafter called *Synechocystis*) was grown in BG-11 medium supplemented with streptomycin and spectimomycin when harboring the self-replicating vectors. The final antibiotic concentrations were Sp  = 50 µg ml^−1^ and Str  = 20 µg ml^−1^. To induce the synthesis of the EFE protein in strains bearing the Lac-inducible promoters, 1 mM of IPTG was added to both, *E. coli* and *Synechocystis*. Cells were cultured at 30°C in all cases. The performance of the metal inducible promoters was measured in modified BG-11 media lacking the appropriate metal to ensure maximum repression. The induction of heterologous protein synthesis was carried out by the addition of 0.5 µM of CuSO_4_ for cells harboring pDF-pet-EFEh, 6 µM of CoCl_2_ for cells harboring pDF-coa-EFEh, or 2 µM of ZnCl_2_ for cells harboring pDF-smt-EFEh. All the cyanobacteria cultures were routinely grown in 250 ml Erlenmeyer flasks at 30°C in a 1% CO_2_ enriched atmosphere with continuous shaking and a light intensity of ≈100 µE m^−2^ s^−1^.

**Table 4:** Strains obtained in this work. Plasmids and strains obtained in this work and their relative ethylene production. n.d. indicates that no ethylene was detected.

Plasmid	*E. coli* construct	*E. coli* ethylene	*Synechocystis* construct	*Synechocystis* ethylene	Promoter
pDF-trc	√	n.d.	√	n.d.	P_trc_
pDF-trc-EFEh	√	+++	√	+++	P_trc_
pDF-lac-EFEh	√	+++	√	+++	P_A1lacO-1_
pDF-pet-EFEh	√	+	√	++	P_petE_
pDF-coa-EFEh	√	+	√	++	P_coa_
pDF-smt-EFEh	√	+	√	+	P_smt_
pDF-lac-ACS-ACO	√	++ *	√	n.d.	P_A1lacO-1_
pDF-luxRI-EFEh	√	+	√	+	P_lux_
pDF-rhlRI-EFEh	√	n.d.	X		P_rhl_

+ indicates a detectable but low rate of ethylene production (<10 nl/ml/h). ++ indicates a medium production (10 to 100 nl/ml/h). +++ indicates a high ethylene production (>100 nl/ml/h). * ethylene was detected only in non-standard conditions e.g. with the addition of substrates (see text for details). X indicates that the construct was not evaluated in *Synechocystis*.

**Figure 1:**
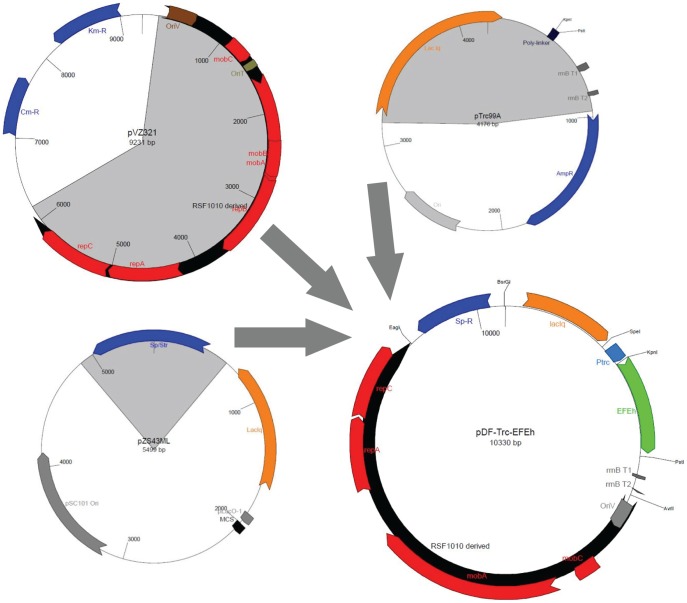
Work-flow for construction of the primary self-replicating wide-host-range vector pDF-trc. The unique restriction sites (Table 1) for exchange of different genetic elements (Table 2) are shown in red.

### 1.2 Codon optimization and gene synthesis

The amino acid sequence of the ethylene forming enzyme from *Pseudomonas syryngae* pv. *phaseolicola*, GenBank accession D13182.1, was synthesized by ATG:biosynthetics (Germany) with the following modifications: DNA sequence was optimized for the preferred codon usage in *Synechocystis*; Six histidine residues (His-tag) were introduced in the protein N-terminal right after the first methionine codon; the repeated sequence cgatg was avoided; and selected unique restriction sites used for plasmid construction were avoided (table 1). For the plant pathway, the *Arabidopsis thaliana* polypeptide sequences ACS7 (UniProt ID Q9STR4) and ACO3 (UniProt ID O65378) were combined as a synthetic operon where each open reading frame was preceded by a ribosomal binding site. The construct was synthesized by GenScript (USA) with the following modifications: codon optimized for *Synechocystis*, and avoiding the selected unique restriction sites used for plasmid construction (Table 1).

**Figure 2:**
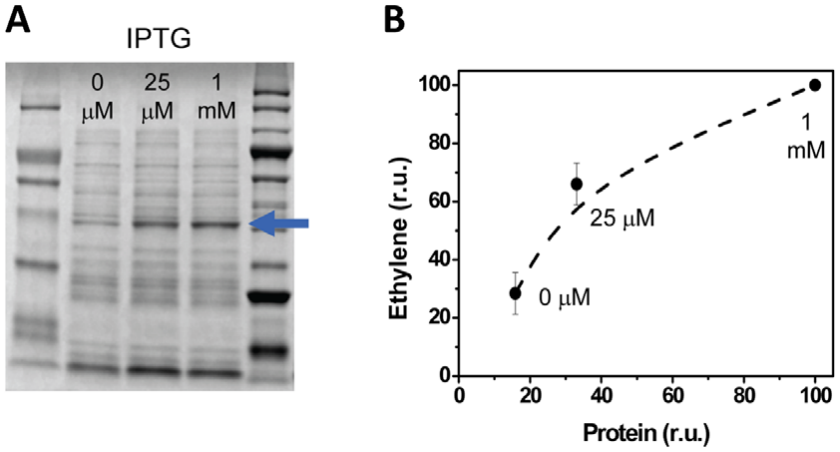
The relationship between the amount of EFE and accumulation of ethylene in the headspace of closed cultures of *E. coli*. *E. coli* DH5α harboring the pDF-trc-EFEh vector with P_trc_ was cultivated in LB medium and induced with various concentrations of added IPTG. (A) EFEh was purified by affinity chromatography and the amount of target protein was quantified using Image Lab software (BioRad) relative to the total protein content and the final OD_600_ of the cultures. (B) The ethylene accumulation in closed *E. coli* DH5α cultivation vessels is plotted relative to the amount of recombinant EFE (in percentage relative to the amount of EFEh with maximum dose of IPTG) that was present in each vessel. Protein synthesis was induced by the addition of 25 µM or 1 mM IPTG that was added at an optical density of 0.1 (600 nm). Cultures to which no IPTG was added were used as controls. The concentration of IPTG that was added to each sample is shown in both panels.

The LuxRI synthetic quorum-sensing system was synthesized (GenScript, USA) using the protein sequences of *Vibrio fischeri* LuxR (UniProt ID P12746) and LuxI (UniProt ID P12747) as template, optimizing the DNA sequence for *Synechocystis*, and removing the restriction enzymes recognition sites shown in Table 1. For the RhlRI system the protein sequences of *Pseudomonas aeruginosa* PAO1 RhlR (UniProt ID P54292) and RhlI (UniProt ID P54291) were used for synthesis (GenScript, USA) with the same modifications as LuxRI. In both constructs the intergenic regions were not modified from the native sequences.

**Figure 3:**
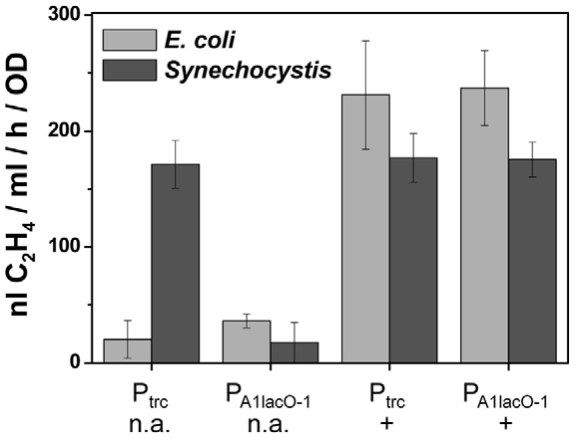
The rate of ethylene synthesis in *E. coli* and *Synechocystis* in response to promoter choice and IPTG. Two different plasmids were evaluated, pDF-trc-EFEh (P_trc_), pDF-lac-EFEh (P_A1lacO-1_). The + symbol indicates that expression was induced by the addition of 1 mM IPTG. Cultures to which no IPTG was added are indicated by n.a.

### 1.3 Construction of the self-replicating wide-host-range pDF-series vectors

The general strategy to construct this set of vectors was to work in “functional blocks” (Table 1) which could be exchanged easily due to the presence of unique restriction sites flanking them. To achieve this, different genetic elements from previously available vectors (Table 2) were either digested and ligated using standard molecular biology procedures, or cloned by PCR using primers (Table 3) designed specifically to generate the appropriate restriction site. When DNA was synthesized (GenScript, USA; ATG:biosynthetics, Germany) the constructs were designed to carry the appropriate restriction sites in the borders.

**Figure 4:**
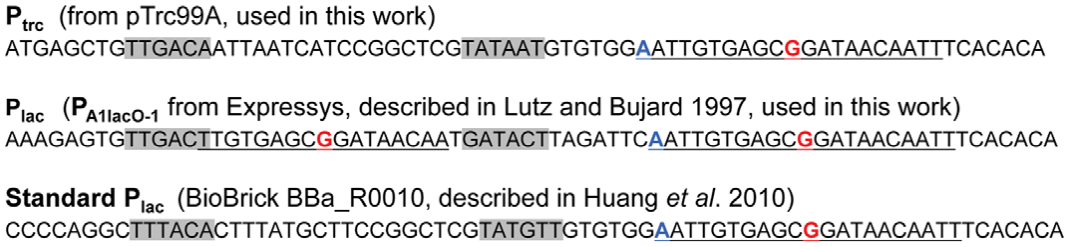
Topography of the lac derived promoters. The -10 and -35 hexamers are highlighted in grey, and the lac operators are underlined. The predicted center of each operator is highlighted in red font. The transcriptional start site [35] is highlighted with bold font and blue color.

**Figure 5:**
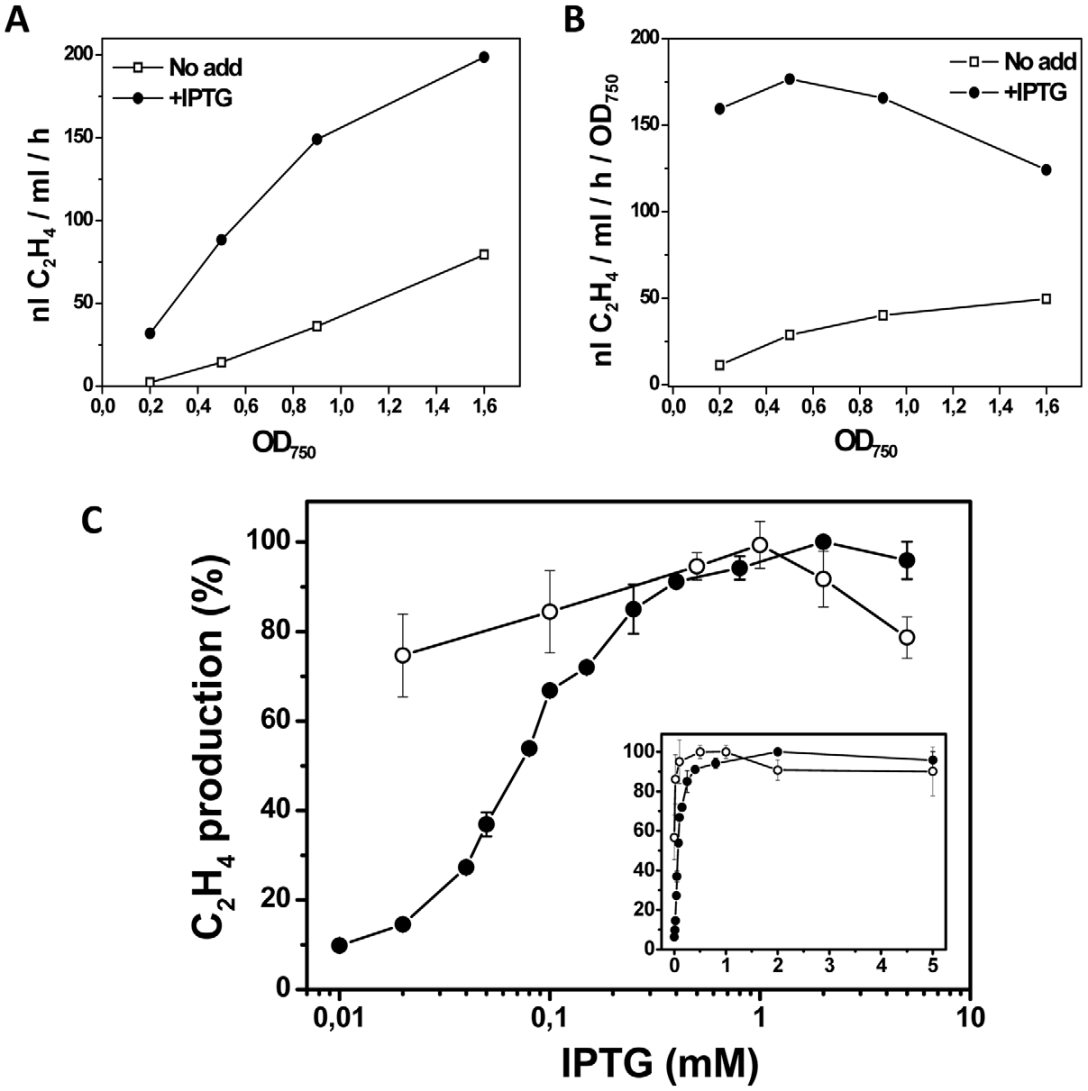
The effect of IPTG concentration, promoter and cell density on the relative rate of ethylene synthesis in *Synechocystis*. (A) The rate of ethylene synthesis with pDF-lac-EFEh (P_A1LacO-1_) in response to the optical density of the culture and the presence or absence of IPTG. Open squares: No IPTG addition. Closed circles: 1 mM IPTG. (B) is identical to (A) with the exception that the rate of ethylene synthesis is shown relative to the optical density. (C) The rate of ethylene synthesis in response to IPTG concentration (logarithmic scale) with pDF-trc-EFEh (P_trc_, black closed symbols), pDF-lac-EFEh (P_A1lacO-1_, white open symbols). All cultures were measured at approximately the same optical density (OD_750_ = 0.5). The inset graph differs only from the larger graph by having a linear scale on the X-axis.

The primary plasmid, named pDF-trc, was constructed by fusion of a DNA region containing the LacI^q^ repressor, Trc promoter, poly-linker and transcription terminators derived from pTrc99a with the self-replicating region from the broad-host range plasmid pVZ321 (a RSF1010 derivative). The antibiotic resistance marker (Sp/Str) of pZE13-MCS (Expressys, Germany) was used to replace the existing selection marker. Finally, the codon-optimized *efe*-gene from *Pseudomonas syringae* (encoding an N-terminal 6-His tag) was inserted into the poly-linker. Additional vectors were constructed from this base plasmid by exchange of the promoter region and/or gene(s) of interest (Table 4).

**Figure 6:**
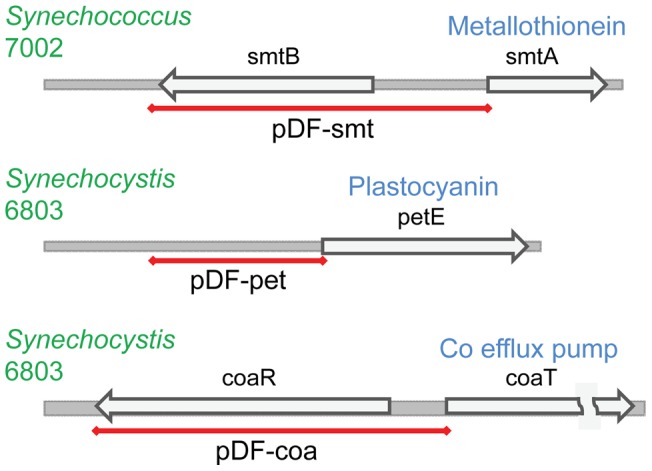
The genomic structure of the native metal-inducible promoter elements. The red line indicates the approximate fragment cloned for the construction of each of the pDF plasmids. The names of the generated vectors are indicated below each red line.

**Figure 7:**
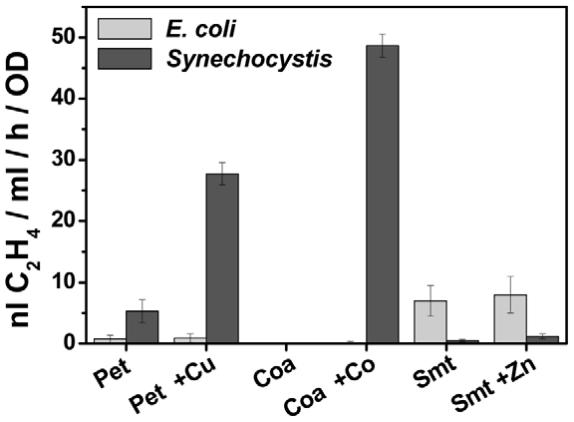
The rate of ethylene synthesis in *E. coli* and *Synechocystis* using three different metal-inducible promoters from cyanobacteria. The rate of ethylene synthesis was analyzed in cultures of *E. coli* (light grey bars) and *Synechocystis* (dark grey bars) carrying the metal-inducible plasmids pDF-pet-EFEh (P_petE_), pDF-coa-EFEh (P_coa_) and pDF-smt-EFEh (P_smt_). Cyanobaterial cultures were grown in BG-11 medium, lacking copper or cobalt for the evaluation of P_petE_ and P_coa_, respectively. *E. coli* cells were grown in LB medium. The ethylene concentration was measured in closed vials that were either not induced (no addition) or induced using 0.5 µM of CuSO_4_ for cells harbouring pDF-pet-EFEh, 6 µM of CoCl_2_ for cells harbouring pDF-coa-EFEh, or 2 µM of ZnCl_2_ for cells harbouring pDF-smt-EFEh.

### 1.4 Transformation of cyanobacteria

Cyanobacteria were transformed either by natural transformation [30] or electroporation [31] performed in a BioRad Gene Pulser electroporation system at 900 V, 125 Ω and 50 µF.

**Figure 8:**
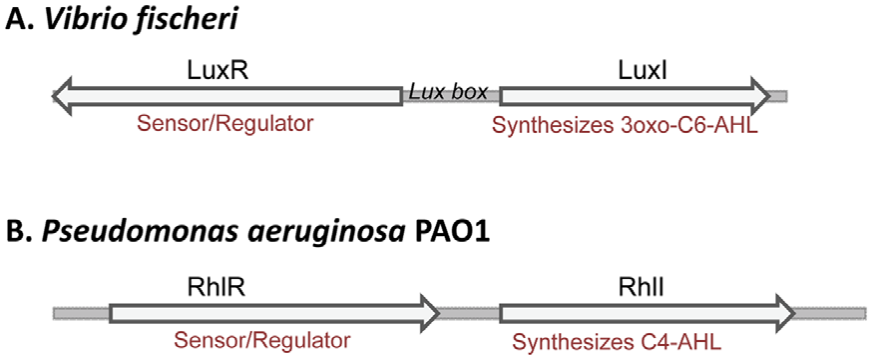
The structure of the synthetic quorum-sensing induction systems used for the constructs (A) pDF-luxRI-EFEh and (B) pDF-rhlRI-EFEh.

### 1.5 Ethylene evolution measurement

*In vivo* measurements in both *E. coli* and cyanobacteria were carried out in cultures in the exponential growth phase. A 1 ml culture was incubated in a 10 ml serum bottle, sealed with a butyl rubber stopper, and incubated at 30°C in a rotary shaker (with growth light in the case of cyanobacteria). Gas samples were extracted from the head-space (25 to 250 µl) with a gas-tight syringe and injected in to a gas chromatograph with flame ionization detector (Perkin Elmer). Samples were separated on a CP-CarboBOND fused silica capillary column from Varian (L = 50 m x ID = 0.53 mm x OD = 0.75 mm). The injection and oven temperature were 80°C, and detector temperature 200°C. Ethylene eluted at approximately 3.5 minutes using helium as the carrier gas at a flow rate of 7 ml min^−1^. A mixture of 99% (v/v) N_2_ with 1% (v/v) C_2_H_4_ was used as a reference standard. Ethylene evolution values are expressed as the mean and standard deviation of at least three replicate cultures. To allow direct comparison between the different strains the ethylene production value was normalized against the optical density of the culture (600 nm for *E. coli*, 750 nm for *Synechocystis*).

**Figure 9:**
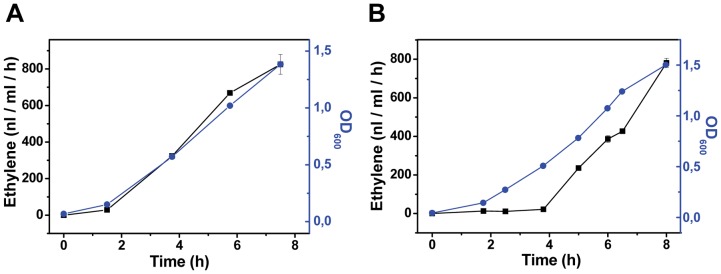
The accumulation of ethylene (black line) and optical density (blue line) with either the (A) constitutive P_trc_ or (B) quorum-sensing P_luxRI_ promoters in *E. coli*. Note that there is effectively no ethylene synthesis until OD_600_ has reached approximately 0.5 with the quorum-sensing promoter. 10 mM FeCl_3_ was added to the LB media in all cultures.

### 1.6 Protein purification

To purify the recombinant His-tagged EFE protein from *E. coli*, 0.3 ml of overnight LB-grown precultures were used to inoculate 15 ml of LB supplemented with different concentrations of IPTG. Cultures were incubated during 5 hours at 30°C. After incubation, 3 ml of each culture was used to measure ethylene evolution, while the remaining culture was pelleted and stored at −20°C. Frozen pellets were thawed and resuspended in a lysis buffer (20 mM Na_2_HPO_4_, 500 mM NaCl, 20 mM imidazole, 1 mg ml^−1^ lysozyme). After the addition of an EDTA-free protease inhibitor cocktail (Roche) cells were sonicated three times for 30 s. Lysate was clarified centrifuging 5 mins at 17000 g to pellet insoluble debris. Supernatant was used to purify the recombinant His-tagged EFE protein using the His SpinTrap™ kit (GE Healthcare) following the manufacturer recommendations. Protein content was quantified using Image Lab software (BioRad).

## Results

### 1.1 Expression of EFE in *Synechocystis* sp. PCC 6803 results in stable synthesis of ethylene

*Synechocystis* harbors a gene (slr1212) annotated as a (putative) ethylene-receptor that has been shown to directly bind ethylene [32] and regulate phototaxis in response to UV-light [33]. Given the reported genetic instability of *Synechococcus elongatus* PCC 7942 transformed with EFE [3], we first confirmed that the addition of ethylene, at a level sufficient to influence a signaling event (1% (v/v) ethylene), to the headspace of closed cultures of *Synechocystis* did not influence the growth and absorption spectra (400–750 nm) over a 3 day period (data not shown). A series of constructs harboring two different ethylene pathways under the control of a diverse set of promoters were thereafter prepared in a broad-host range RSF1010 plasmid (hereafter named pDF) from a primary vector bearing the Trc promoter (Figure 1). All final plasmid constructs were confirmed to be correct by DNA sequencing. Following transformation of both *E. coli* DH5α and *Synechocystis*, ethylene accumulation was observed in the headspace of closed cultures of most strains. No ethylene synthesis could be observed in some strains (noted in Table 4) despite repeated attempts to transform the host and confirmation of successful transformation by PCR and/or plasmid isolation (and verification by sequencing or diagnostic restriction digestion). Plasmids were maintained in both hosts throughout the project period. For example *Synechocystis* strain transformed with plasmid pDF-trc-EFEh (harboring P_trc_ and codon-optimized EFE with N-terminal His-tag) was in all cases stable and produced ethylene with a similar rate of evolution when maintained for more than 6 months in liquid culture. The addition of 10 µM iron (as FeCl_3_) tripled the rate of ethylene synthesis in *E. coli*, although no such effect was observed in cyanobacteria. Omission of the EFE-encoding gene resulted in no measurable accumulation of ethylene in either of the two hosts. The addition of six histidine residues to the N-teminal end of EFE did not influence ethylene-synthesis (data not shown).

### 1.2 Using EFE as a non-invasive promoter-reporter to identify systems for well-regulated expression in *Synechocystis*

In order to utilize EFE as a promoter-reporter, we first confirmed that the rate of ethylene synthesis correlated with the amount of recombinant EFEh protein in *E. coli* using P_trc_ (pDF-trc-EFEh) which is an established tunable promoter [34]. The accumulation of ethylene was measured and recombinant His-EFE protein was thereafter purified by nickel-affinity chromatography and quantified following SDS-PAGE (Figure 2). The protein content correlated with the accumulation of ethylene in the headspace suggesting that total EFE activity is the main rate-limiting factor for ethylene synthesis in *E. coli*. This confirms that EFE-dependent ethylene synthesis can be used as a facile and non-invasive promoter-reporter. The relative degree of control and promoter strength of a series of promoters was thereafter evaluated. The studied promoters could be divided into three groups: (1) Lac-derived (IPTG induced) promoters, (2) native cyanobacterial metal inducible promoters, and (3) synthetic quorum-sensing promoters.

#### 1.1 1. Lac-derived promoters

A commonly used *E. coli* promoter (P_trc_) was initially tested in both *E. coli* and *Synechocystis*. In *E. coli* the repression in the absence of IPTG was effectively complete (Figure 3). The transformation of *Synechocystis* with the same plasmid resulted in approximately the same rate of ethylene accumulation compared to *E. coli* and the presence or absence of IPTG only had a minor impact on ethylene synthesis. The rate of ethylene synthesis observed in *Synechocystis* was similar to that previously described when the native EFE gene was expressed in *Synehococcus elongatus* PCC 7942 [3] and *Synechocystis* [4].

Since repression of the P_trc_ in *Synechocystis* was poor, in accordance with earlier studies [18], further promoters were tested including a variant of the Lac promoter, P_A1lacO-1_ [35] (Figure 4). Initial experiments with P_A1lacO-1_ showed surprisingly strong repression and similar rates of ethylene synthesis as with P_trc_ (Figure 3). Upon closer inspection it was found that the degree of repression in the absence of IPTG related to the optical density of the culture at the time of ethylene-measurement (Figure 5a, 5b), with tight repression in low-density cultures and progressively weaker repression the more dense the culture became. This prompted a re-evaluation of the repression afforded by P_trc_ in low-density cultures; however, the superior control with the P_A1lacO-1_ promoter was still evident (Figure 5c).

#### 1.1.2. Native metal-inducible promoters from cyanobacteria

The few systems described up to date in the literature that are able to carry out finely tuned heterologous expression in cyanobacteria are native metal-inducible promoters, including promoters preceding genes encoding plastocyanin (*petE* [36]), cytochrome *c*6 (*petJ* [37]), the nrsBACD operon [38], CoaT (*coaT* [38]) and metallothionein (*smtA* [39]). In order to compare the relative utility of these promoters with the *E. coli* promoters, we selected three of these systems (P_petE_, P_coa_, P_smt_ – Figure 6), sub-cloned them into the self-replicating plasmid in place of the lacI^q^ and P_trc_ elements and evaluated all constructs in both *E. coli* and *Synechocystis* (Figure 7). Not surprisingly, the cyanobacterial promoters performed poorly in *E. coli*. In cyanobacteria the repression afforded by the metal-inducible promoters was much better than with P_trc_ in the absence of inducing conditions, although the rate of ethylene synthesis was at best 25% compared with the strong lac-derived promoters. The relatively poor regulation observed with P_petE_ is probably due to the difficulty in removing all residual copper in standard water and laboratory glassware. Interestingly P_smt_ from *Synechococcus* sp. PCC 7002 allowed stronger induction of protein synthesis in *E. coli* compared to *Synechocystis*, with only residual ethylene evolution activity detected in the latter host (Figure 7, Table 4).

#### 1.1.3. Synthetic quorum-sensing promoters

It has been suggested that quorum-sensing promoters have evolved to enable “communication” within microbial communities [40]. This is achieved with a two-component system where the first component encodes the protein that catalyzes the synthesis of the chemical autoinducer (N-acyl homoserine lactone), and the second component acts as sensor and regulator (Figure 8). In theory a self-regulated system may be useful in order to avoid the addition of costly chemicals to induce gene expression in cultures that cannot utilize constitutive promoters. There are no studies, as far as we are aware, that have attempted to utilize orthogonal synthetic quorum-sensing based promoters in cyanobacteria.

We designed two synthetic quorum-sensing systems (LuxRI and RhlRI, Table 2 and 4) and placed them in front of the His-tagged *efe* gene in the pDF vector (Figure 8). LuxRI from *Vibrio fischerii* is induced by 3-oxo-hexanoyl homoserine lactone [41], and RhlRI from *Pseudomonas aeruginosa* is induced by butanoyl homoserine lactone [42]. Both constructs were used to transform both *E. coli* and cyanobacteria and evaluated for ethylene synthesis. In *E. coli*, the LuxRI construct functioned as expected, with ethylene accumulation only being observed after the culture reached a threshold cell density (OD_600_ ≈ 0.5, Figure 9). The rate of ethylene synthesis was similar between strains harboring the P_trc_ and P_luxRI_ constructs, suggesting that the pDF-LuxRI-EFEh construct was functional. In *Synechocystis*, however, only a low rate of ethylene synthesis (≈10 nl C_2_H_4_ ml^−1^ h^−1^) was observed independent of cell density (data not shown). The presence of the plasmid in the cyanobacterial cells was confirmed by PCR, together suggesting that the LuxRI promoter as designed in the present study did not function as expected. Constructs carrying the *Pseudomonas aeruginosa* RhlRI promoter did not function in *E. coli* DH5α (rate of ethylene synthesis <5 nl C_2_H_4_ ml^−1^ h^−1^, data not shown) at different cell densities, even after external addition of the inducer butanoyl homoserine lactone. The construct was therefore not tested in cyanobacteria.

### 1.3 Alternative ethylene pathway

Li and coworkers [43] demonstrated that over-expression of the two key-genes in the plant ethylene biosynthesis pathway, ACC oxidase (sourced from tomato) and ACC synthase (sourced from soybean), as a fusion product in *E. coli* enabled the synthesis of ethylene *in vitro* using crude extracts fed with S-adenosyl-L-methionine. We attempted to reconstruct the plant pathway *in vivo* by co-expression of the two individual proteins in *E. coli* designed with *Arabidopsis thaliana* genes as template. No ethylene accumulation was observed under standard conditions used with EFEh. Supplementation of the medium with 5 mM bicarbonate, 5 mM sodium ascorbate and 10 µM FeCl_3_ resulted in a production of 30 nl ethylene ml^−1^ h^−1^. In the cyanobacterium *Synechocystis* no activity was detected with or without supplementation.

## Discussion

In previous studies, when the EFE-pathway was introduced to *Synechococcus elongatus* PCC 7942, a rapid loss of catalytic function was repeatedly observed through targeted mutation of the encoding gene [3]. In contrast, no loss of activity was observed in the present study in *Synechocystis* despite repeated sub-culturing, allowing EFE-dependent ethylene synthesis to be used as a reporter for the evaluation of promoter constructs. During the preparation of this manuscript another study appeared that also demonstrated stable ethylene evolution in *Synechocystis* using a codon-optimized *efe* gene [4]. What is the reason for this contrast in genetic stability between the two organisms? The two previous studies ([3] and [4]) both utilized light-induced promoters and chromosomal integration of the *efe* gene, while in the present study we employed orthogonal promoters to express an optimized *efe* gene in a self-replicating plasmid system. Interestingly, under similar conditions, the ethylene evolution in all three studies was in the range of 200 nl C_2_H_4_/ml culture/hour. As it appears that neither the promoter nor the system used to express the gene (chromosomal integration *versus* plasmid-based) have an influence on the ethylene evolution stability, we may conclude that the instability observed in the first study ([3]) is caused by: (1) host strain, and/or (2) *efe* codon optimization. Although it is not possible to exclude any one of these factors, the mutations in the *efe* gene introduced to *Synechococcus elongatus* PCC 7942 were repeatedly located in the same region of the gene. This region contained repeated sequences that were removed by codon optimization in our study. Further investigation will be necessary to resolve this outstanding question.

The Lac derived promoters enabled the greatest rates of ethylene synthesis in cyanobacteria in the present study. Previously, GFP-expression with Trc and Lac promoters were compared in *Synechocystis* [18]. It was concluded that the Trc promoter was strong but not regulated, whilst the Lac promoter was very weak. In contrast, we found that with only moderate variation in the promoter structure (Figure 4), a variant of the Lac promoter (P_A1lacO-1_) enable strong protein expression with fine-tuned regulation, although the degree of control became progressively more relaxed the higher the density of the cultures became. Since strong repression was observed with P_A1lacO-1_, the lack of repression with some of the Lac promoters is unlikely to be due to the functionality of the Lac repressor protein in *Synechocystis*.

The difference in repression between P_trc_ and P_A1lacO-1_ is most likely due to the presence of a second lac operator sequence in the P_A1lacO-1_ promoter between the -35 and the -10 region (Figure 4, operator regions are underlined). The apparent enhanced competitiveness of LacI^q^ with P_A1lacO-1_ may then be explained by either (1) an increased chance of binding, assuming that LacI^q^ binding at either of the two sites will negatively influence the binding of the sigma factor, and/or (2) the additional operator is more favorably positioned to negatively influence binding by the sigma factor.

It is also possible that the difference in regulation between these promoters may be caused by the variation in the structure of the -35 and -10 regions. P_trc_ possesses the “standard” bacterial structure TTGACA-17n-TATAAT, while P_A1lacO-1_ has a TTGACT-17n-GATACT structure. It is therefore possible that the relevant *Synechocystis* sigma factors display different selectivity for these two promoter regions, resulting in differing expression of the EFE protein and consequently different rates of ethylene biosynthesis.

The gradual relaxation in EFE-expression at increasing culture density that is observed with P_A1lacO-1_ in the absence of IPTG is unfortunate but interesting. A possible explanation is that endogenous sugars, such as allolactose, may accumulate in high-density culture cells and bind to LacI^q^. Alternatively, the distribution of sigma factors may change in response to cell culture density, in turn influencing binding to the promoter-region and/or competition with the LacI^q^ repressor.

The metal-inducible promoters P_petE_ and P_coa_ functioned well in *Synechocystis* as demonstrated previously [38]. They provide a useful complement to the IPTG-inducible promoters, although the utility of P_petE_ may be compromised by the difficulty in completely removing copper from laboratory glassware and water. In addition, as the repressor for the PetE promoter is unknown it was not included in the plasmid constructs. Therefore, the weak repression observed with the PetE promoter may also have been caused by an imbalance between PetE and its corresponding repressor. In contrast, the repression of the transcription using P_coa_ in a cobalt-depleted BG-11 medium was excellent, even without extra precautions in preparation of the media. In the case of P_smt_ it is not a surprise that this system from *Synechococcus* sp. PCC 7002 does not function in *Synechocystis*, taking into account that *Synechocystis* lacks the gene for metallothionein. Nevertheless, *Synechocystis* contains a zinc exporter – *ziaA* – that is controlled by an SmtB-like repressor (*ziaB*) [44]. In addition, the upstream region of the zinc response gene *ziaA* shows some homology with the sequence upstream of the *Synechococcus* sp. PCC 7002 metallothionein gene. Still, these similarities are not sufficient to make the heterologous P_smt_ system function in *Synechocystis*.

The lack of induction in cyanobacteria with the synthetic quorum-sensing systems LuxRI and RhlRI may be explained by a lack of readily available substrate (acyl-ACP and S-adenosyl-*L*-methionine) or incompatibility between the native sigma factors and the heterologous promoter regions from which the LuxRI and RhlRI regions were sourced. In addition, RhlRI is in *Pseudomonas aeruginosa* associated with the LasRI system in a manner not fully understood yet [45]. It is therefore possible that also factors other than substrate are missing. The addition of commercial homo-serine lactones from multiple suppliers was also tried without effect, most likely reflecting the instability of the compounds since GC-MS analysis repeatedly failed to yield any distinct peak with the expected mass spectra (data not shown).

The ACS-ACO pathway strains did not produce ethylene in neither of the two tested prokaryotes even though ethylene synthesis earlier was reported with a recombinant synthetic ACS-ACO fusion [43]. Closer inspection of the work of Li and coworkers [43] indicate that only minute quantities of ethylene were obtained with crude extracts of the synthetic ACS-ACO fusion and no information regarding *in vivo* synthesis in *E. coli* cultures were provided. There are several possible reasons for the lack of activity in the present study: (1) unfortunate choice of gene-source, in turn influencing protein synthesis and/or activity with available substrate, (2) lack of stabilizing ACS factors present in native environment [46], (3) lack of pyridoxal phosphate (PLP) as a cofactor, (4) inactivation of ACO by Cobalt present in standard BG-11 media. Given that ethylene synthesis was observed upon addition of substrate to *E. coli* whole cells, and no ethylene was observed with negative control cells that harbored the empty pDF-trc vector, the lack of functional recombinant ACS and ACO could at least be discarded. Given that the EFE-pathway functioned well and the ACS-ACO pathway is expected to also generate HCN as an undesirable by-product, we did not further examine the cause of the lack of function with the ACS-ACO pathway.

## Conclusions

We recommend the P_A1lacO-1_ promoter for use in *Synechocystis* in cases when reasonably well-regulated and potentially strong protein expression is desired, although it will be important to monitor and control culture density for reproducible outcome. If lower protein expression levels are acceptable, the metal-inducible promoters such as P_coa_ will be able to offer effectively complete repression in the absence of inducing conditions. Since stable production of ethylene in cyanobacteria is now possible with *Synechocystis*, this opens the road for the establishment of photo-biotechnological systems for direct conversion of sunlight, CO_2_ and water into this highly versatile industrial chemical. However, further optimization of the host and cultivation system will most likely be needed to enable economically sustainable production systems.
